# Digital Twin of an Optical Measurement System

**DOI:** 10.3390/s21196638

**Published:** 2021-10-06

**Authors:** Michiel Vlaeyen, Han Haitjema, Wim Dewulf

**Affiliations:** Department of Mechanical Engineering, KU Leuven, Celestijnenlaan 300, 3001 Leuven, Belgium; han.haitjema@kuleuven.be (H.H.); wim.dewulf@kuleuven.be (W.D.)

**Keywords:** digital twin, laser line scanner, coordinate measuring machine

## Abstract

Digital twins of measurement systems are used to estimate their measurement uncertainty. In the past, virtual coordinate measuring machines have been extensively researched. Research on digital twins of optical systems is still lacking due to the high number of error contributors. A method to describe a digital twin of an optical measurement system is presented in this article. The discussed optical system is a laser line scanner mounted on a coordinate measuring machine. Each component of the measurement system is mathematically described. The coordinate measuring machine focuses on the hardware errors and the laser line scanner determines the measurement error based on the scan depth, in-plane angle and out-of-plane angle. The digital twin assumes stable measurement conditions and uniform surface characteristics. Based on the Monte Carlo principle, virtual measurements can be used to determine the measurement uncertainty. This is demonstrated by validating the digital twin on a set of calibrated ring gauges. Two validation tests are performed: the first verifies the virtual uncertainty estimation by comparison with experimental data. The second validates the measured diameter of different ring gauges by comparing the estimated confidence interval with the calibrated diameter.

## 1. Introduction

According to the Guide to the Expression of Uncertainty in Measurement (GUM), every measurement needs to be accompanied with an uncertainty, otherwise, a measurement is considered incomplete [1]. The measurement uncertainty can be estimated by either a Type A or Type B evaluation. The International Vocabulary of Metrology (VIM) defines Type A evaluation as: “Evaluation of a component of measurement uncertainty by a statistical analysis of measured quantity values obtained under defined measurement conditions [2]”. This uncertainty, Type A, can be derived from a series of independent repeated observations. A Type B evaluation is defined as: “Evaluation of a component of measurement uncertainty determined by means other than a Type A evaluation of measurement uncertainty [2]”. This uncertainty, Type B, is determined using the available information, such as: previous measurement data, manufacturer’s specifications, calibration data and experience [3,4]. Both types are characterized by a standard deviation.

The uncertainty determination for measurements of complex geometries is challenging for both types of evaluation. Type A evaluation is time-consuming when multiple measurements need to be performed. Since the manufacturing industry is evolving towards shorter inspection times and higher quality, a bottleneck, in order to estimate the uncertainty, is to be avoided [5]. When a large batch of complex geometries is measured, the manufacturer opts for a Type B evaluation. However, if the geometries are unique, a reliable Type B evaluation is not always possible. A solution to this challenge is a virtual measurement device, a digital twin of its physical counterpart. The digital twin generates virtual measurements, while the physical machine can continue its operations. This allows an uncertainty evaluation including a Type A evaluation without creating a bottleneck in the production process.

The digital twin mimics the behavior of its physical counterpart, considering the specified error contributors. The measurement uncertainty is the result of multiple contributors, such as the environment, the measurement device, the sampling strategy, the evaluation strategy and the work piece [6,7]. Based on a Monte Carlo method, multiple virtual measurements are performed in order to determine the measurement uncertainty. Digital twins of coordinate measuring machines (CMMs) have been extensively researched in the past [6,8,9,10,11,12,13,14,15,16]; however, research into digital twins of optical systems is still lacking due to the high number of error contributors [17]. This paper presents a method to obtain and simulate a digital twin of a laser line scanner (LLS) mounted on a CMM.

## 2. Determination of the Measurement Error

The considered measurement system consists of three mayor hardware components, namely the CMM, the articulating probe head and the LLS. The CMM positions and orientates the optical sensor, i.e., the LLS, relative to the inspected object, while the LLS generates a point cloud of the measured points on the surface of the object. The total error of the measurement system is the summation of the errors of both systems separately. The error contribution of the CMM is described in literature and is used in this paper to simulate the error of the CMM [10,12]. The error contribution of the sensor is based on systematic and random error models for specified surfaces in function of the measurement strategy [18].

### 2.1. Error Contribution from the CMM

The most notable error contributors of the CMM are the hardware errors. Therefore, the presented digital twin focuses on the geometric errors. These geometric errors of a CMM are determined by external factors, i.e., temperature, humidity and vibrations, and internal factors, such as rotations, misalignments, straightness of the guideways and scale errors. However, the external factors are indirectly integrated in the model. As a consequence the digital twin is only valid in a temperature controlled environment with a small temperature gradient or drift as a result of internal heating. A. Gąska et al. presented a virtual CMM for the uncertainty estimation of coordinate measurements performed in industrial conditions [16]. A conventional CMM has three axes of movement and each movement involves six components of deviation: three linear and three angular. The errors on the perpendicularity between the three axes are characterized by three squareness errors. Considering the CMM as a rigid body, the geometric errors can by described with 21 parametric errors [6,12].

**Figure 1:**
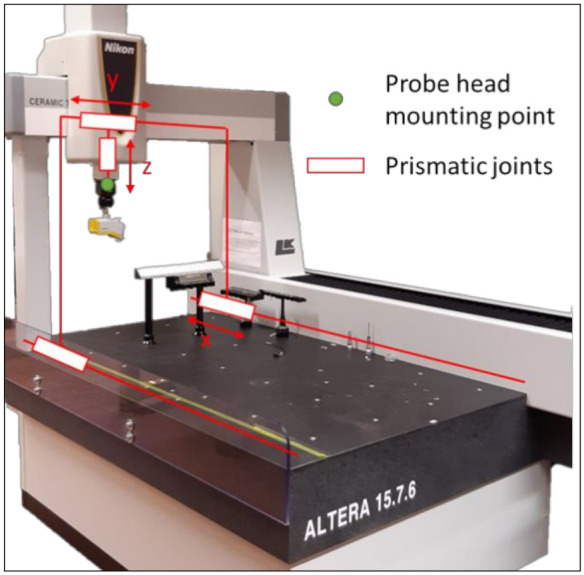
A CMM with the prismatic joints which allows three translation movements of the phmp along the axis of movement. X, Y and Z denote the linear movement.

The kinematic model of the CMM consists of four rigid bodies connected by three prismatic joints, allowing three degrees of freedom for the probe head mounting point (phmp), i.e., translations in the directions of the main axes [12]. The literature [12,19,20] describes the position of the phmp as (1) and the total angular error of phmp as (2) with respect to the coordinate system of the CMM. Figure 1 depicts the components of the CMM.

(1) p0phmp=xenc+exx+exy+exz−yencecx+zencebx+ebyyenc+eyx+eyy+eyz−zenceax+eayzenc+ezx+ezy+ezz+yenceax

(2) R0phmp=1−ecz−ecy−ecxebz+eby+ebxecx+ecy+ecz1−eaz−eay−eax−ebx−eby−ebzeax+eay+eaz1

$x_{enc}$, $y_{enc}$ and $z_{enc}$ represent the values read from linear encoder of the scales. The error components for a straight line motion along an axis are represented with $e\lambda_{1}\lambda_{2}$, where $\lambda_{1}$ represents the type of kinematic error and $\lambda_{2}$ represents the axis. For example, the six kinematic error components along the x axis in function to the position on that axis are:

- *exx*: positioning error of the x axis;
- *eyx*: straightness error motion of the x axis in the y direction;
- *ezx*: straightness error motion of the x axis in the z direction;
- *eax*: roll error motion of the x axis;
- *ebx*: pitch error motion of the x axis due to a rotation around the y axis;
- *ecx*: yawt error motion of the x axis due to a rotation around the z axis.

The kinematic errors can be described by equations similar to (3). The linear and quadratic function represent the linear and quadratic component of the kinematic error, while the harmonics correspond with the higher-order undulations of the kinematic error with *N* the maximum number of undulations per length *L* of the axis. For example for *exx* can be written:

(3) $$exx\left(x\right)=s\left(\frac{2x}{L_{x}}-1\right)+c\left(6{\left(\frac{x}{L_{x}}\right)}^{2}-\frac{6x}{L_{x}}+1\right)+\sum_{n=1}^{N}a_{n}\cos\left(2n\pi x/L_{x}+\phi_{n}\right)$$

for $x\in \left[0,L\right]$ and with:

- *s* the coefficient of the linear term;
- *c* the coefficient of the quadratic term;
- *a_n_* the coefficients describing the higher *n*-th order order undulations of the geometric deviations;
- *ϕ_n_* the phase of the higher order undulations $\phi_{n}\in \left[0,2\pi \right]$.

In (3), the first two terms are the shifted Legendre polynomials of the first and second degree and the remaining terms are the Fourier coefficients that describe the higher order undulations of the geometrical deviations. It is taken for granted that there is some dependence between the Legendre polynomial coefficients and the Fourier coefficients [21]; still, this is considered as an appropriate way of expressing the geometrical errors [12]. In (3), the zero-order (constant) term is omitted as length measurements are always based on differences between points, and the constant term in the pitch, yaw and roll motions representing the squareness is already taken into account by the linear term in the straightness errors.

Disregarding the correlation between *s* and *c* on one hand, and the *a_n_* coefficients on the other hand, the standard deviation of the errors can be approximated by:

(4) $$\sigma \left(e\left(x\right)\right)\approx \sqrt{\frac{1}{3}s^{2}+\frac{1}{5}c^{2}+\sum_{n=1}^{N}\frac{a_{n}^{2}}{2}}$$

For the expressions of the various geometrical errors the following considerations can be made:

*exx*, *eyy* and *ezz* represent the scale errors where the linear term will be dominant as this contains the effects of temperature and thermal expansion of the object and the scales. The other terms represent calibration and production errors of the scales and these are usually small. A known periodic error that may occur is due to the interpolation that will have a periodicity of once and/or half the scale pitch, which is typically 20 µm or 8 µm [22]; this effect is disregarded here. An estimate for the typical errors arising can be made from the temperature range and the uncertainty of the expansion coefficient of the scales and steel objects. The maximum error will be about 2 × 10^−6^·*L,* where *L* is the measured length, which gives *s* = 2 × 10^−6^ at maximum for *exx*, *eyy* and *ezz*.

*eyx*, *ezx*, *exy*, *ezy*, *exz* and *eyz* represent the straightness errors. The linear term *s* in these errors represent the squareness errors. The orientation of the coordinate system may be defined by putting *s*(*eyx*), *s*(*ezx*) and *s*(*ezy*) equal to zero, then *s*(*exy*), *s*(*ezy*) and *s*(*eyz*) represent the three squareness errors of the CMM. Their magnitude may be estimated from explicit squareness measurements or from the CMM specifications.

For the nine roll errors it may be stated that they have a zero linear term as the squareness errors are already incorporated in the linear term of the straightness representation, and not all are relevant, depending on the CMM configuration. As the roll errors are caused by straightness deviations of the mechanical guidances, there is a correlation between the straightness and roll. In general this correlation depends on the mechanical construction of the guidances and will be complicated for higher order undulations. Still, for low-order undulations, especially the quadratic term, it can be assumed that the rotary error is the derivative of the straightness: *s*(*ecx*) * = 6·c*(*eyx*)*/L*, *s*(*eby*) = *6·c*(*ezx*)*/L* and *s*(*eay*) = *6·c*(*ezy*)*/L*. It is assumed that rotation-errors have no higher-order terms so there is only an s parameter.

The error contribution of the CMM is estimated by assuming the geometrical errors of the CMM ‘as they could be’. With this ‘virtual CMM’ a measurement is taken from which a parameter is calculated; e.g., a measurement of a cylinder is simulated from which the diameter is estimated. This is repeated for CMM having a different error structure by recalculating the geometrical errors ‘as they could also be’ and repeating this same measurement using the newly calculated error structure, giving another cylinder measurement from which another diameter is calculated. Repeating this for many different error structures gives a distribution of cylinder diameter values, from which conclusions can be drawn about the distribution of cylinder diameter values in terms of systematic and random deviations from the nominal cylinder diameter value.

A virtual CMM is constructed by randomizing the geometric errors by putting random factors in (3) as following:

(5) $$exx\left(x\right)=k\cdot s\left(\frac{2x}{L_{x}}-1\right)+k\cdot c\left(6{\left(\frac{x}{L_{x}}\right)}^{2}-\frac{6x}{L_{x}}+1\right)+\sum_{n=1}^{N}a_{n}\cos\left(\frac{2n\pi x}{L_{x}}+m\cdot 2\pi \right)$$

where the random factor *k* varies every time it appears in the 18 equations similar to (5) by which a virtual CMM is constructed, according to a beta distribution with the first shape factor 2.8 and the second shape factor 5.2. The beta distribution is shifted by minus 1.25 and multiplied by a random sign (6). These parameters result in a distribution with two peaks around ±1. As a result each individual geometric error is more likely to be represented realistically in the total error contribution, rather than a marginal contribution, which is the case with a normal distribution around 0. To represent each individual geometric error realistically with a normal distribution, the standard deviation has to be chosen so large that the virtual CMM cannot conform to its specifications. The random number *m* is taken from a uniform, rectangular distribution between 0 and 1. For every simulation the parameters *s*, *c* and an are given realistic values; see the considerations above and [12]. Keeping the *a_n_* parameters constant while varying the phase randomly keeps the autocorrelation function of the periodic errors constant which enables a proper uncertainty estimation as was shown in [23].

The random number *k* is taken from a Beta-distribution as following:

(6) $$k\in \pm \left(\mathrm{Beta}\left(2.8,5.2\right)-1.25\right)$$

The simulated CMM has an MPE (maximum permissible error) of 1.8 μm + 2.5 × 10^−6^·*L*. Such a CMM must be simulated in such a way that this limit is kept. This is accomplished by considering this limit as a 95% confidence interval that is approximated by two standard uncertainties. Estimating linear, length-dependent terms *s* can be accomplished by taking *s* in *exx*, *eyy*, *ezz*, *exy*, *ezy* and *eyz* equal to 1.25 × 10^−6^/2√2, considering that the scale linearity and the squareness contribute independently to an equal extent.

For the constant term of 1.8 µm it can be considered that this limit must be kept in three directions (*x*, *y* and *z*). Assuming a standard uncertainty of 1.8 µm/2 and six independent contributions in each direction this is achieved by putting a limit of 0.9 µm/√6 = 0.36 µm to the standard uncertainty imposed by the coefficients *c* and *a_n_*. (4) is used to estimate realistic values of the parameters c and *a_n_* where the total contribution to the standard uncertainty of parameter c on one hand and the parameters an on the other hand is taken equal for the straightness components. The parameters *a_n_* are chosen from a normal distribution with mean of 0 and a standard distribution of 1 with *N* equal to 15. The random values are scaled so that (7) is valid.

(7) $$\sqrt{\sum \frac{a_{i}^{2}}{2}}=0.2\;µm$$

**Figure 2:**
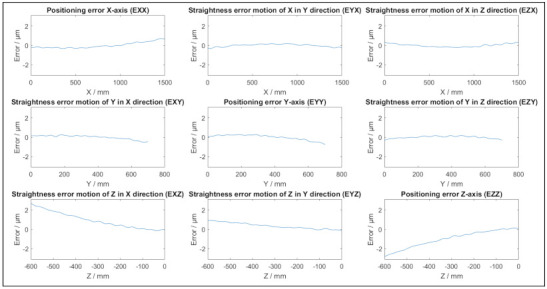
Example of positional and straightness errors of a single simulation of a virtual CMM.

**Figure 3:**
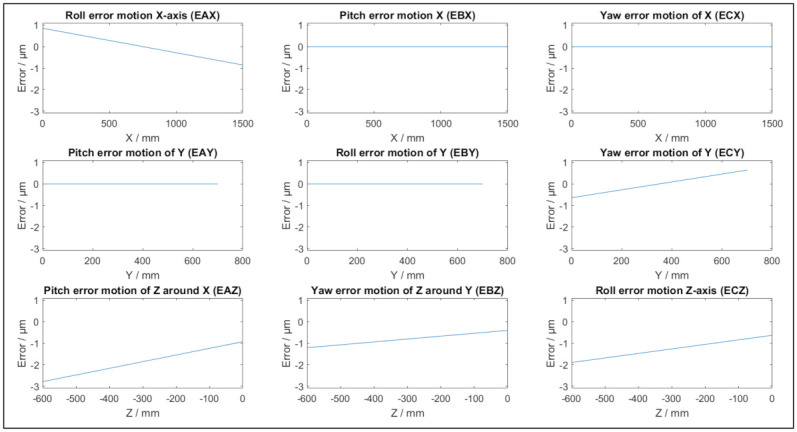
Example of angular errors of a single simulation of a virtual CMM.

**Figure 4:**
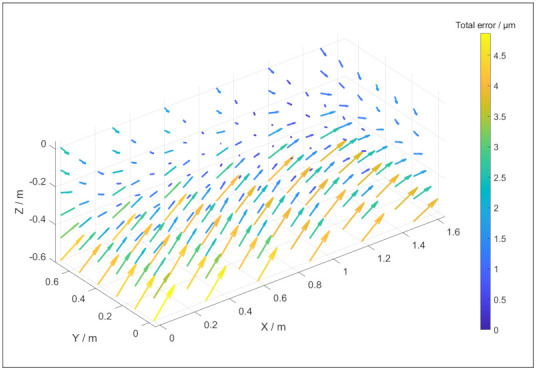
An example of the total error of the phmp in the coordinate system of a virtual CMM. The error components of this virtual CMM are depicted in Figure 3 and Figure 4.

Figure 2 depicts the positional and straightness errors of a potential virtual CMM based on Table 1 and (5). The parameters are randomly chosen with a maximum of 15 undulations per length. Figure 3 depicts typical angular errors of the virtual CMM as they appear in this specific single simulation, with the parameters in Table 2. The total error as a function of the position in the coordinate system of the simulated CMM is depicted in Figure 4. The size and direction of the error is determined by (5), and due to the random parameter *k* different simulations result in different error propagations. From Figure 2 and Figure 3 it can be conducted that each individual error is smaller than 3 μm, yet the magnitude of the maximal total error is approximately 5 μm, as displayed in Figure 4. As an example, a length measurement over the entire Y axis starting in the origin is considered. The error in the origin is negligible. The error on the other end of the Y axis is 3 μm in the negative Y direction. As a result, the length measurement will appear 3 μm shorter than the true value.

In order to validate the virtual CMM, its conformance to ISO 10360-2 is verified. This acceptance test has a statistical nature; as a consequence false rejections and false acceptances are inherent to this method. Recently, a method has been presented to manage the risk involved in the conformance test [24]. Since the parameters of the virtual CMM are validated based on various tests and thus the statistical nature of the acceptance test is negligible, the conventional ISO 10360-2 is used. This standard has three objectives [6]. For the first and second objective, it measures the error on a calibrated test length, with and without any probe offset. Therefore, the virtual machine, just like the physical counterpart, must have an error lower than the given MPE. The acceptance test of this standard can be performed on a gauge block, a step gauge or a ball bar. There should be five different lengths measured in the seven different directions of the CMM. The smallest length should be at least 30 mm and the longest length should be 66% of the total measurable length of the orientation. The third objective of ISO 10360-2 is to test the repeatability of a measurement. Therefore, the standard specifies that each measurement is repeated three times [25].

**Figure 5:**
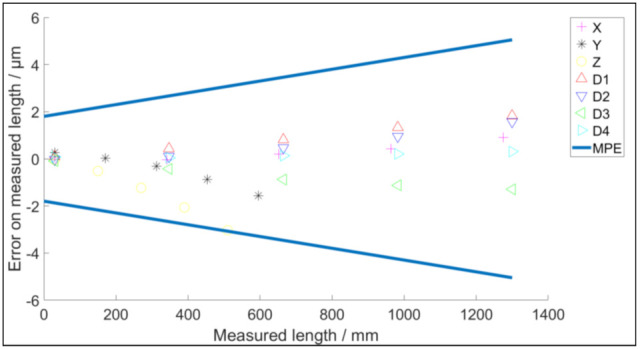
ISO 10360−2 test for the virtual CMM.

Figure 5 depicts the simulated results of the acceptance test according to ISO 10360-2 for the virtual CMM with the error map depicted in Figure 4. Once a virtual CMM is generated based on the 18 geometric errors, no random error is involved in the determination of the positional error as a function of the phmp’s position in the coordinate system. Therefore, the repeatability of the virtual CMM is not tested at this stage; when incorporating the optical probe it will be inherently added. The simulated measurements are performed along the three main axes of the CMM, namely X, Y and Z. The four diagonals are labelled D1, D2, D3 and D4. When all measurements are within the limits of the MPE of the CMM, which is 1.8 μm + 2.5 × 10^−6^·*L*, the virtual machine passes the acceptance test. The performance indicator *v* (8) for the digital twin is calculated in order to validate the chosen parameter in Table 1 and Table 2 [12].

(8) v=minL mpeL err, for all measured lengths

**Figure 6:**
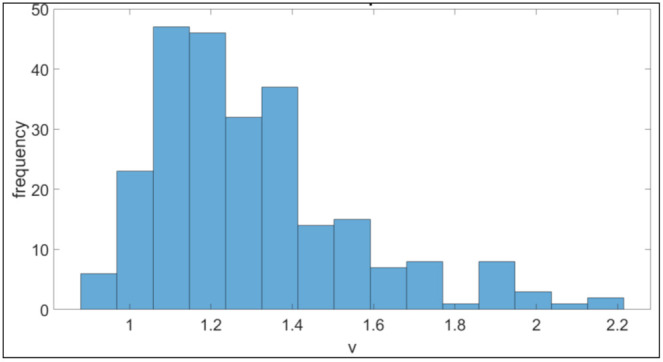
250 performance indicators of the virtual CMM based the ISO 10360-2 acceptance test.

The error on the measured length is *^err^L* and the MPE for the corresponding length is *^mpe^L*. If the performance indicator is less than 1, the virtual CMM fails the acceptance test. A performance indicator larger than 2 represents a virtual CMM that is twice as accurate as the MPE indicates. This means that the parameters used for the simulation are not realistic. For the validation of the parameters of Table 1 and Table 2, 250 acceptance tests are performed on different virtual CMMs. The performance indicators are depicted in Figure 6. 95% of the simulated CMMs pas the acceptance test, proving the feasibility of the parameters of Table 1 and Table 2. Based on the performance indicators in Figure 6, it can be concluded that the simulated CMMs show realistic measurement errors which are not too accurate.

### 2.2. Articulating Probe Head

**Figure 7:**
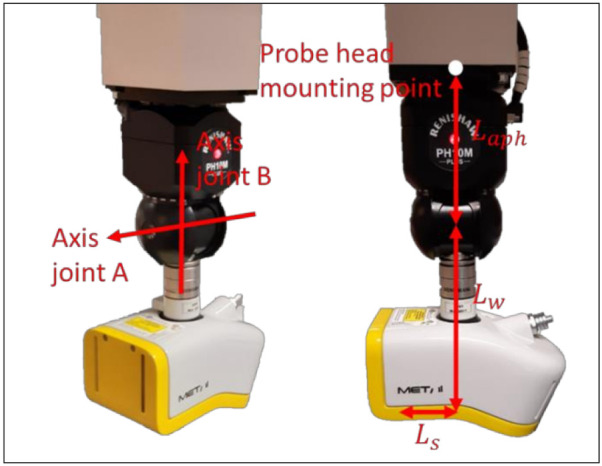
The articulating probe head with an LLS as probing system mounted on it.

The articulating probe head, Figure 7, allows the probe system to measure from different directions. It has two joints: joint A controls the elevation in the vertical plane and joint B controls the direction in the horizontal plane; both joints are depicted in Figure 7 [26]. The joints are incremental and are asynchronous with the movement of the CMM. The probing system cannot change its orientation and position simultaneously. Therefore, the degrees of freedom of a CMM-controlled measurement system are often referred to as 3 + 2 degrees of freedom. ISO 10360-5 covers the verification of the articulating probe head [27].

The probe head error involves the error introduced by the articulating probe head. The simulated articulating probe head error is Renishaw PH10M. From the specifications, a repeatability (2 s) of 0.5 μm at a distance of 100 mm is derived [28]. In the digital twin this is simulated by taking a standard deviation of 2.5 × 10^−6^ rad, (9), as rotational error for both joint A and B, which gives a repeatability of 0.5 μm at a distance of 100 mm.

(9) $$\tan\left(\frac{0.25\;\mu m}{100\;\mathrm{mm}}\right)=2.5\times 10^{-6}\;\mathrm{rad}$$

Although this rotational error is small, it can result in a significant error due to the distance of the probing system relative to the measured point. The rotation matrix determined by the joints of the articulating probe head, is expressed as a function of the angle made by joint A, *θ_a_*, and joint B, *θ_b_*, the respective errors on each joint are *E_a_* and *E_b_*. Equation (10) gives the rotation matrix used to orientate the probing system without error. After each manipulation of the articulating probe head the error needs to be calculated. A random number of a normal distribution with a standard deviation in accordance with the technical specifications, is added to the angle of the manipulated joint. Equation (11) expresses the rotation matrix including the random error. Any offset error on a joint is neglected, since the calibration of the articulating probe head compensates the offset errors.

(10) R0aph=cosθacosθb−sinθb−sinθacosθbcosθasinθbcosθb−sinθasinθbsinθa0cosθa

(11) R0apherr=cosθa+Eacosθb+Eb−sinθb+Eb−sinθa+Eacosθb+Ebcosθa+Easinθb+Ebcosθb+Eb−sinθa+Easinθb+Ebsinθa+Ea0cosθa+Ea

### 2.3. Probing System: Laser Line Scanner

**Figure 8:**
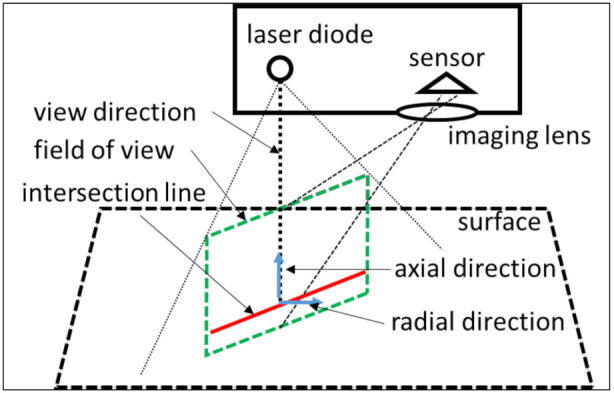
Triangulation principle of an LLS based on [18].

The probing errors are dependent on the selected measurement system. The selected measurement system is an LLS. The LLS projects a laser line on an object. The laser beam’s reflection on the surface of the measurand is captured by the sensor, i.e., photodetector, of the LLS. Based on triangulation, the scanner determines the point of intersection between the projected laser beam and the surface (Figure 8).

Digital twins, simulating tactile probing errors, often use a normal distributed random error as a representation of the error contributors [12]; for optical probes this task is more complex. The error contributors of an optical probing system can be classified in multiple categories [7,18,29,30,31,32]:

- Environmental error sources: temperature, humidity, ambient light, dust and vibrations;
- Surface properties of the object: colour, reflectivity, surface finishing and translucency;
- Measurement strategy: position of probing system relative to the object and object’s position in the CMM;
- Hardware of the probing system: accuracy of components and mechanical instabilities;
- Data processing: resolution, filtering and algorithms;
- Extrinsic factors: operators, surface cleanliness and clamping system.

**Figure 9:**
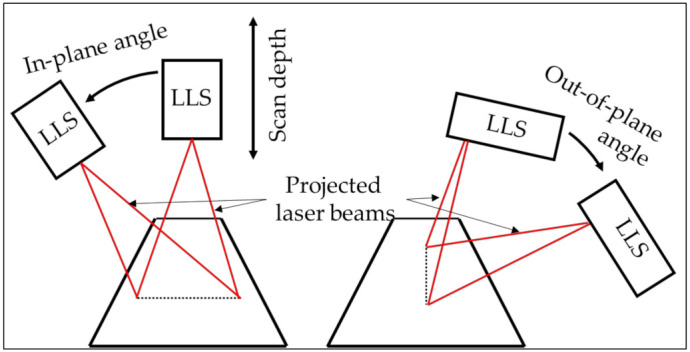
Scan depth, in-plane angle and out-of-plane angle of an LLS based on [32].

The variety of error contributors makes it difficult to generate a digital twin of an optical probing system which incorporates all these error contributors. Therefore, some assumptions are considered for the presented digital twin of an optical probing system. First of all, the presented digital twin represents a physical counterpart that operates in a controlled environment, i.e., the environmental error sources are minimized. Secondly, the influence of the hardware of the probing system and extrinsic factors are neglected. Thirdly, the proposed model to simulate the error propagation of an LLS is valid for specified surface characteristics and sensor settings. The model expresses the systematic error and the random error as second degree polynomial, Equations (12) and (13), as a function of the scan depth, in-plane angle and out-of-plane angle. These parameters are depicted in Figure 9.

(12) $$E_{sys}=c_{1,s}d^{2}+c_{2,s}d\alpha_{out}+c_{3,s}d\alpha_{in}+c_{4,s}d+c_{5,s}\alpha_{out}^{2}+c_{6,s}\alpha_{out}\alpha_{in}+c_{7,s}\alpha_{out}+c_{8,s}\alpha_{in}^{2}+c_{9,1}\alpha_{in}+c_{10,s}$$

(13) $$E_{rand}=c_{1,r}d^{2}+c_{2,r}d\alpha_{out}+c_{3,r}d\alpha_{in}+c_{4,r}d+c_{5,r}\alpha_{out}^{2}+c_{6,r}\alpha_{out}\alpha_{in}+c_{7,r}\alpha_{out}+c_{8,r}\alpha_{in}^{2}+c_{9,r}\alpha_{in}+c_{10,r}$$

with:

- *E_sys_*: the systematic error;
- *E_rand_*: the random error;
- *d*: the scan depth;
- *α_in_*: the in-plane angle;
- *α_out_*: the out-of-plane angle;
- *c*_(1–10,*s*)_: the coefficients for the systematic error model;
- *c*_(1–10,*r*)_: the coefficients for the systematic error model.

The scan depth is the distance between the point on the inspected surface, *P*, and the laser source, *L_S_*.

(14) $$d=\sqrt{{\left(LS_{x}-P_{x}\right)}^{2}+{\left(LS_{y}-P_{y}\right)}^{2}+{\left(LS_{z}-P_{z}\right)}^{2}}$$

The angle between the incoming laser beam, $\vec{v}$, and the normal on the inspected surface, $\vec{n}$, can be split up in two components: in-plane angle and out-of-plane angle. The in-plane angle is the component in the field of view. The out-of-plane angle is the component perpendicular to the field of view (Figure 9). The in-plane angle is expressed by (15) and the out-of-plane angle by (16) [18]. The scan direction of the LLS is $\vec{m}$ in these equations.

(15) $$\alpha_{in}=\left\{\begin{array}{c} if\;\arccos\left(\frac{\left(\vec{n}-\frac{\vec{n}\cdot \left(\vec{m}\times \vec{v}\right)}{{\left\|\vec{n}\right\|}^{2}}\left(\vec{m}\times \vec{v}\right)\right)\cdot \vec{m}}{\left\|\vec{n}-\frac{\vec{n}\cdot \left(\vec{m}\times \vec{v}\right)}{{\left\|\vec{n}\right\|}^{2}}\left(\vec{m}\times \vec{v}\right)\|\|\vec{SE}\right\|}\right)\geq 90{}^{\circ}:-\arccos\left(\frac{\left(\vec{n}-\frac{\vec{n}\cdot \left(\vec{m}\times \vec{v}\right)}{{\left\|\vec{n}\right\|}^{2}}\left(\vec{m}\times \vec{v}\right)\right)\cdot \left(-\vec{m}\right)}{\left\|\vec{n}-\frac{\vec{n}\cdot \left(\vec{SE}\times \vec{v}\right)}{{\left\|\vec{n}\right\|}^{2}}\left(\vec{SE}\times \vec{v}\right)\|\|-\vec{m}\right\|}\right)\\ if\;\arccos\left(\frac{\left(\vec{n}-\frac{\vec{n}\cdot \left(\vec{m}\times \vec{v}\right)}{{\left\|\vec{n}\right\|}^{2}}\left(\vec{m}\times \vec{v}\right)\right)\cdot \left(\vec{m}\times \vec{v}\right)}{\left\|\vec{n}-\frac{\vec{n}\cdot \left(\vec{m}\times \vec{v}\right)}{{\left\|\vec{n}\right\|}^{2}}\left(\vec{m}\times \vec{v}\right)\|\|\vec{m}\right\|}\right)<90{}^{\circ}:\arccos\left(\frac{\left(\vec{n}-\frac{\vec{n}\cdot \left(\vec{SE}\times \vec{v}\right)}{{\left\|\vec{n}\right\|}^{2}}\left(\vec{SE}\times \vec{v}\right)\right)\cdot \left(-\vec{m}\right)}{\left\|\vec{n}-\frac{\vec{n}\cdot \left(\vec{SE}\times \vec{v}\right)}{{\left\|\vec{n}\right\|}^{2}}\left(\vec{SE}\times \vec{v}\right)\|\|-\vec{m}\right\|}\right) \end{array}\right.$$

(16) $$\alpha_{out}=\left\{\begin{array}{c} if\;\arccos\left(\frac{\left(\vec{n}-\frac{\vec{n}\cdot \vec{m}}{{\left\|\vec{n}\right\|}^{2}}\vec{m}\right)\cdot \left(\vec{m}\times \vec{v}\right)}{\left\|\vec{n}-\frac{\vec{n}\cdot \vec{m}}{{\left\|\vec{n}\right\|}^{2}}\vec{m}\|\|\vec{m}\times \vec{v}\right\|}\right)\geq 90{}^{\circ}:-\arccos\left(\frac{\left(\vec{n}-\frac{\vec{n}\cdot \vec{m}}{{\left\|\vec{n}\right\|}^{2}}\vec{m}\right)\cdot \left(-\vec{m}\right)}{\left\|\vec{n}-\frac{\vec{n}\cdot \vec{m}}{{\left\|\vec{n}\right\|}^{2}}\vec{m}\|\|-\vec{m}\right\|}\right)\\ if\;\arccos\left(\frac{\left(\vec{n}-\frac{\vec{n}\cdot \vec{m}}{{\left\|\vec{n}\right\|}^{2}}\vec{m}\right)\cdot \left(\vec{m}\times \vec{v}\right)}{\left\|\vec{n}-\frac{\vec{n}\cdot \vec{m}}{{\left\|\vec{n}\right\|}^{2}}\vec{m}\|\|\vec{m}\times \vec{v}\right\|}\right)<90{}^{\circ}:\;\arccos\left(\frac{\left(\vec{n}-\frac{\vec{n}\cdot \vec{m}}{{\left\|\vec{n}\right\|}^{2}}\vec{m}\right)\cdot \left(-\vec{m}\right)}{\left\|\vec{n}-\frac{\vec{n}\cdot \vec{m}}{{\left\|\vec{n}\right\|}^{2}}\vec{m}\|\|-\vec{m}\right\|}\right) \end{array}\right.$$

**Figure 10:**
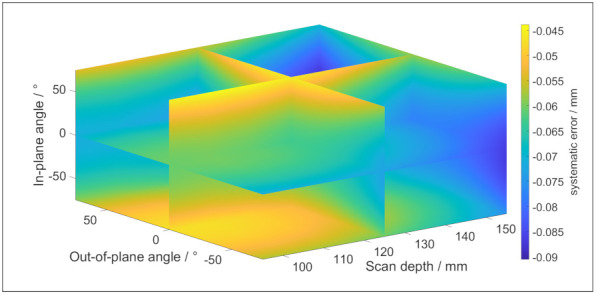
The systematic error of the LLS for stainless steel surfaces in function of the measurement strategy.

**Figure 11:**
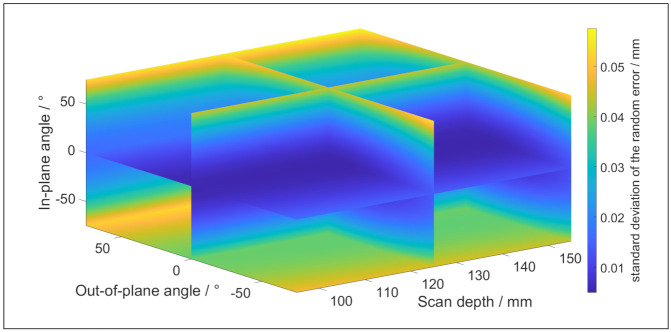
The random error of the LLS for stainless steel surfaces in function of the measurement strategy.

The coefficients of (12) and (13) are determined by scanning a reference sphere [18]. The values of each coefficient can be found in Table 3 and Table 4, and the settings of the scanner are presented in Table 5. These coefficients are valid for a stainless surface, such as the reference sphere from which they are obtained and the artefacts which are used for the validation. Figure 10 and Figure 11 depict respectively the systematic error and the random error according to (12) and (13).

### 2.4. Articulating Probe Head

The total measurement error of a point is the summation of the error induced by the CMM, articulating probe and the LLS. In order to calculate the error, the position of the laser source, the movement direction of the LLS and the normal on the surface need to be determined. Since an LLS travels in a straight line from the start position to the end position while scanning, the vector of the movement direction is the difference of the end position and the start position. When performing a virtual measurement with a digital twin, the input of the object is a virtual geometry, such as an STL model or a CAD model. An STL model provides information of the normal on each facet, which can be used to calculate the in-plane angle and out-of-plane angle. For a basic three-dimensional geometrical object, the normal can be calculated without a virtual model. For example, for a sphere, the normal on the surface in a specified point is the vector from the center to the specified point. The position of the laser source can be obtained by (17) [18]. This equation represents the intersection of the plane containing the laser beams and the scan trajectory with start position, *S*.

(17) $$\left\{\begin{array}{c} LS_{x}\\ LS_{y}\\ LS_{z} \end{array}\right\}=\frac{\left(\left\{\begin{array}{c} P_{x}\\ P_{y}\\ P_{z} \end{array}\right\}-\left\{\begin{array}{c} S_{x}\\ S_{y}\\ S_{z} \end{array}\right\}\right)\cdot \vec{m}}{{\vec{m}}^{2}}\cdot \vec{m}+\left\{\begin{array}{c} S_{x}\\ S_{y}\\ S_{z} \end{array}\right\}$$

The axes of joint A and B are located at an offset, *L_aph_*, of the phmp. The position of the laser source has also a fixed offset to the intersection of the axes of joint A and B. The position of the laser source relative to the intersection is given by (17) for joint A and B equal to zero.

(18) $$\left\{\begin{array}{c} LS_{x,rel}\\ LS_{y,rel}\\ LS_{z,rel} \end{array}\right\}=\left\{\begin{array}{c} 0\\ -L_{w}\\ -L_{ls} \end{array}\right\}$$

Multiplying (18) with the rotation matrix of the articulating probe head, (10), expresses the relative position in function of the joints.

(19) $$\left\{\begin{array}{c} LS_{x,rel}\\ LS_{y,rel}\\ LS_{z,rel} \end{array}\right\}=\left\{\begin{array}{c} \sin\left(\theta_{b}\right)L_{w}+\sin\left(\theta_{a}\right)\cos\left(\theta_{b}\right)L_{ls}\\ -\cos\left(\theta_{b}\right)L_{w}+\sin\left(\theta_{a}\right)\sin\left(\theta_{b}\right)L_{ls}\\ -\cos\left(theta_{a}\right)L_{ls} \end{array}\right\}$$

Knowing the position of the laser source, the position of the phmp can be determined by going backwards up the kinematic chain.

(20) $$phmp=\left\{\begin{array}{c} LS_{x}\\ LS_{y}\\ LS_{z} \end{array}\right\}-\left\{\begin{array}{c} 0\\ 0\\ -L_{aph} \end{array}\right\}-\left\{\begin{array}{c} \sin\left(\theta_{b}\right)L_{w}+\sin\left(\theta_{a}\right)\cos\left(\theta_{b}\right)L_{ls}\\ -\cos\left(\theta_{b}\right)L_{w}+\sin\left(\theta_{a}\right)\sin\left(\theta_{b}\right)L_{ls}\\ -\cos\left(theta_{a}\right)L_{ls} \end{array}\right\}$$

The error on the probe head mounting point, *E*_phmp_, can be calculated, as explained in Section 2.1, once the coordinates are known. The error, *E_orientation_*, in a measured point as a result of the angular errors of the CMM, (2), and the angular errors of the joint A and B, (10) and (11), is expressed by (21).

(21) Eorientation=R0phmp×R0apherr0−Lw−Lls+v→d−R0phmp×R0aph0−Lw−Lls+v→d

The error introduced by the LLS, *E_LLS_*, can be determined as a function of the scan depth, in-plane angle and out-of-plane angle, as explained in Section 2.3. This error consists of two components: the random error, (13), and the compensated systematic error, (11). However, it is hard to compensate for the systematic error in its entirety. As a result, a remaining uncertainty on the compensated systematic error is introduced. This uncertainty is represented with the factor δ. For the simulations of the validations this factor is 0.01.

(22) $$E_{LLS}=N\left(0,E_{rand}^{2}\right)+N\left(0,{\left(\delta E_{sys}\right)}^{2}\right)$$

The total error can be expressed by (23):

(23) $$E_{tot}=E_{phmp}+E_{orientation}+E_{LLS}$$

A point on the surface, *P_true_*, that is measured by the digital twin can be simulated as a measured point, *P_meas_*, by making use of (23).

(24) $$P_{meas}=P_{true}+E_{tot}$$

## 3. Virtual Measurement

**Figure 12:**
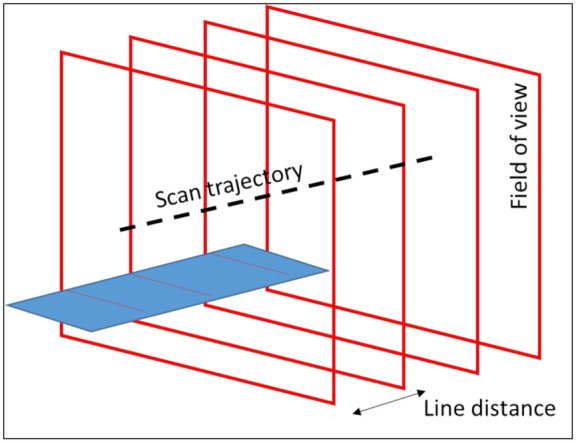
Point cloud generation of a surface (blue) by multiple fields of view.

**Figure 13:**
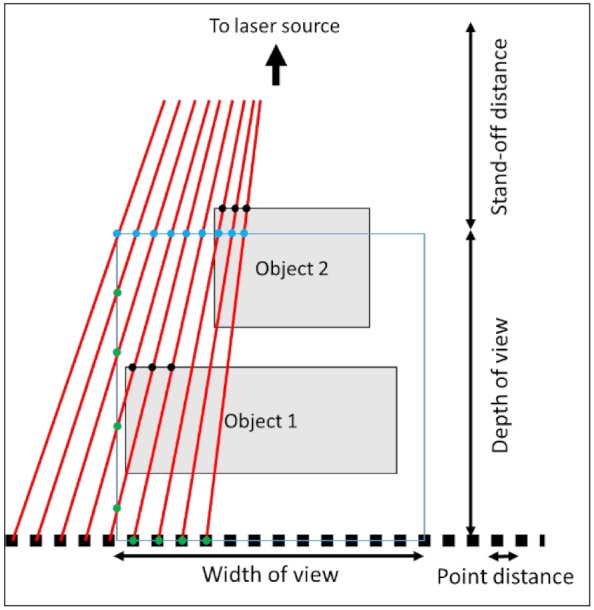
The intersection of the laser beams and the objects in order to determine the measured points.

In order to simulate a virtual measurement the measured points on the digital object need to be determined. Based on the scan trajectory, i.e., the line from the start point to the end point and the orientation of the LLS, the field of view is determined. The measured points are located in this field of view. By combining multiple fields of view, each separated by a specified line distance, a point cloud can be generated (Figure 12). In order to determine the points in the field of view, the possible intersection of a laser beam and the digital object is calculated (Figure 13). The field of view is a 2D square with a depth of view and a width of view at a stand-off distance, i.e., the minimal distance to scan points, of the laser source. At the bottom of the field of view a line of points is created. The points have a uniform distance between each other. A line, which represents the laser beam, goes from the laser source to these points. If this line intersects with the digital model in the field of view, a measured point is established. An example is illustrated in Figure 13. The lines, going from the laser source to the black squares, intersect with two objects. The intersections are the black dots. If the laser beam has multiple intersections with the objects, the intersection closest to the laser is chosen. If this intersection is in the field of view, between the blue and green dots in Figure 13, the point is measured. As can be seen in Figure 13, Object 1 is scanned, while Object 2 is too close to the laser source and cannot be scanned. This process is repeated for each field of view to generate the entire point cloud. If the digital object is an STL model of the file, a ray-triangle intersection algorithm can be used to determine the measured points. However, an STL model is a segmented model of a geometry and thus introduces an error. For simple geometries, such as spheres or cylinders, this error can be compensated. The STL model gives information about the normal on the surface in the measured point, which can be used to determine the in-plane and out-of-plane angle. Based on these parameters, the error on each individual point of the point cloud is determined. For a Monte Carlo simulation, new error values are generated for each simulation. For the uncertainty estimation in this paper, 100 Monte Carlo simulations are generated.

## 4. Validation

**Figure 14:**
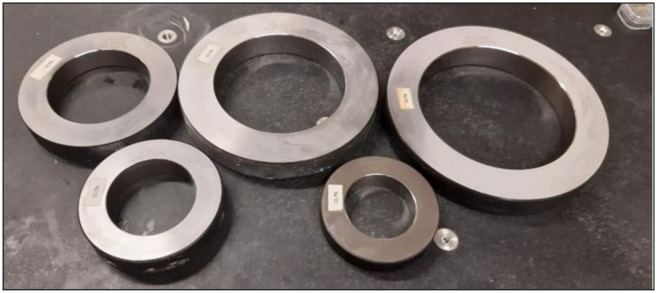
The used ring gauges for the validation of the digital twin.

The validation of the digital twin is performed on a set of ring gauges. The ring gauges used in the experiments are depicted in Figure 14. There are two main reasons to opt for these artefacts. Firstly, the measurement of the inner cylinder activates all the components of the measurement system: the CMM has to move over a significant distance, the articulating probe head has to rotate to measure the different sides of the cylinder and the LLS has to measure a great amount of points. Secondly, the surface characteristics of the artefact which provided the model (12) and (13) are similar to the characteristics of the ring gauges. The experiments are executed with the LLS setting as indicated Table 5. The experiments are performed with Nikon Metrology LC60Dx LLS and an LK Altera 15.7.6 CMM.

Two different types of tests are performed as validation. The first test compares the uncertainty estimation obtained by the digital twin with the uncertainty of the repeated experiments. One hundred repeated measurements are experimentally performed on a ring gauge with a diameter of 125 mm. The objective is to confirm if the digital twin correctly estimates the measurement uncertainty. The second test measures a set of ring gauges with each a different diameter. The digital twin is used to determine the 95% confidence interval of each measurement. The aim is to validate the digital twin by conforming that the calibrated diameters of the ring gauges are within the 95% confidence interval of the measured diameters.

**Figure 15:**
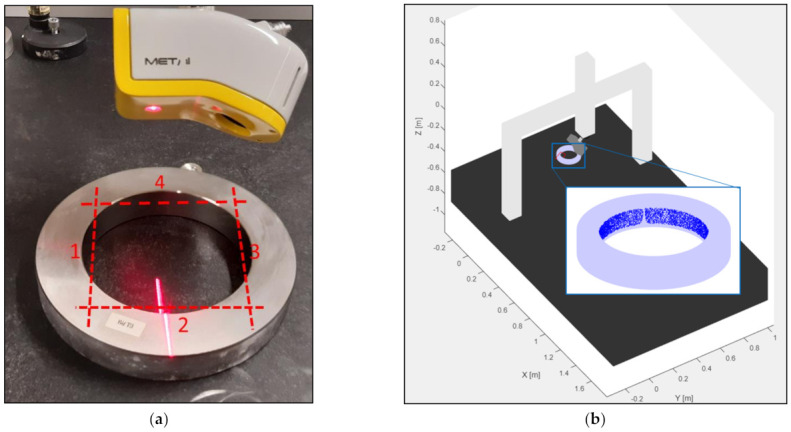
(**a**) The scan trajectories (red dashed lines) of the measurement of the inner cylinder; (**b**) The data (blue dots) of the simulated measurement in the digital environment.

**Figure 16:**
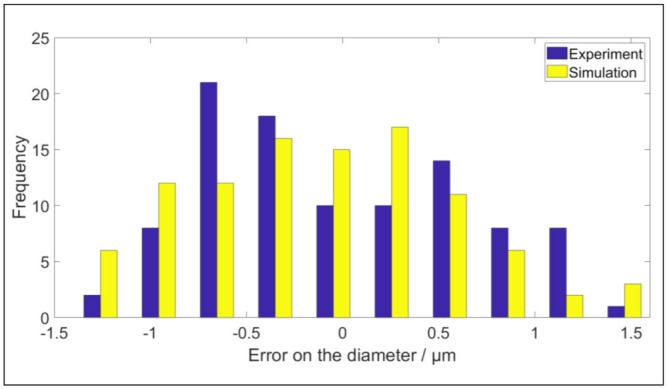
Comparison between the experimentally obtained diameters and the diameters obtained by simulation.

The first test measures a ring gauge with diameter of 125 mm. Four uniformly distributed sides of the inner cylinder are measured. The scan tracks of the four sides are depicted in Figure 15a,b, illustrating the measured data in the simulated environment of the digital twin. In order to reduce the calculation time, the data is selected according to a grid. The grid has 25 rows in the central axis direction of the cylinder and 400 columns on the circumference of the cylinder. The rows and columns are uniformly distributed over the measured cylinder. The measured point closest to the intersection of the gridlines is selected. After this filtration, maximally 10,000 points are used to determine the diameter and to estimate the uncertainty. The systematic error on measured points in both validation tests is compensated [18]. The least squared diameter is determined for each full measurement of the ring gauges. Both the experimental and the simulated diameter are depicted in Figure 16. In this figure it can be seen that the virtual and experimental measurements have a similar uncertainty range. The standard deviation of the diameter for the experimental data is 0.645 µm and for the simulated data it is 0.684 µm. Therefore, it can be concluded that the digital twin correctly mimics the optical measurement system.

The second test measures a set of ring gauges, each with a different diameter. The diameters are 60 mm, 70 mm, 90 mm, 125 mm and 150 mm (Figure 14). Each ring gauge is measured once in a similar way as the first test was performed and the systematic error is compensated according to [18]. The 95% confidence interval is determined by 100 simulations. The simulated measurement results are assumed to be normally distributed. As a consequence the total confidence interval is four standard deviations, i.e., 4 s. The lower confidence level, *LCL*, is expressed by (25) and the upper confidence level, *UCL*, is expressed by (26). The calibrated diameter, the measured diameter, *LCL* and *UCL* can be found in Table 6. The results in this table confirm that the digital twin of the optical system offers a reliable uncertainty estimation for all diameters involved.

*LCL* = measured diameter − 2s (25)

*UCL* = measured diameter + 2s (26)

## 5. Conclusions

This paper presents a method to simulate a digital twin of an optical measurement system. The components of the digital twin, namely the CMM, the articulating probe head and the LLS, are described. The expression of a virtual CMM and articulating probe head is summarized from the state-of-the-art and applied to the presented digital twin. The virtual LLS determines the measurement error in function of the scan depth, in-plane angle and out-of-plane angle. This digital twin assumes stable measurement conditions and uniform surface characteristics. The digital twin is validated on a set ring gauges with each a different diameter. The validation confirms that the digital twin can be used to determine the measurement uncertainty of the complex geometries. This determination can be performed offline in order to shorten inspection times.

## 6. Discussion

Further research is required to include the influence of all the error contributors, such as the influence of the temperature. Furthermore the error model of the LLS is dependent on the surface characteristics of the measurand. To estimate the measurement uncertainty of the object with multiple different surfaces the error model needs to be defined as a function of the surface characteristics. Keeping the ongoing Industry 4.0 evolution in mind, it would be interesting to use ‘Big Data’ to check and alter the parameters of the virtual measurement device. This will ensure that the estimated uncertainty corresponds with the actual situation.

## Figures and Tables

**Table 1:** Parameters used to simulate the positional and straightness errors of Figure 2.

	*s*/10^−6^	*c*/µm	$\sqrt{\sum \frac{a_{i}^{2}}{2}}/µm$	*L*/m
*exx*	0.44	0.37	0.2	1.500
*eyx*	0	0.37	0.2	1.500
*ezx*	0	0.37	0.2	1.500
*exy*	0.44	0.37	0.2	0.700
*eyy*	0.44	0.37	0.2	0.700
*ezy*	0	0.37	0.2	0.700
*exz*	0.44	0.37	0.2	0.600
*eyz*	0.44	0.37	0.2	0.600

**Table 2:** Parameters used to simulate the rotational (angular) angular errors of Figure 3.

	*s*/10^−6^	*L*/m
*eax*	0.8	1.500
*ebx*	0.8	1.500
*ecx*	6·*c*(*eyx*)/*L*	1.500
*eay*	6·*c*(*ezy*)/*L*	0.700
*eby*	6·*c*(*ezx*)/*L*	0.700
*ecy*	0.8	0.700
*eaz*	0.8	0.600
*ebz*	0.8	0.600
*ecz*	0.8	0.600

**Table 3:** The coefficients of the systematic error model of the LLS.

*c* *_*1*,s_*	*c* *_*2*,s_*	*c* *_*3*,s_*	*c* *_*4*,s_*	*c* *_*5*,s_*	*c* *_*6*,s_*	*c* *_*7*,s_*	*c* *_*8*,s_*	*c* *_*9*,s_*	*c* *_*10*,s_*
1/mm	1/rad	1/rad	/	mm/rad^2^	mm/rad^2^	mm/rad	mm/rad^2^	mm/rad	mm
$-6.6922\times 10^{-6}$	$-4.3130\times 10^{-7}$	$8.2636\times 10^{-7}$	$1.3893\times 10^{-3}$	$-1.8983\times 10^{-6}$	$2.5683\times 10^{-7}$	$4.5744\times 10^{-5}$	$2.5560\times 10^{-6}$	$-6.2625\times 10^{-5}$	$-1.3218\times 10^{-1}$

**Table 4:** The coefficients of the random error model of the LLS.

*c* *_*1*,r_*	*c* *_*2*,r_*	*c* *_*3*,r_*	*c* *_*4*,r_*	*c* *_*5*,r_*	*c* *_*6*,r_*	*c* *_*7*,r_*	*c* *_*8*,r_*	*c* *_*9*,r_*	*c* *_*10*,r_*
1/mm	1/rad	1/rad	/	mm/rad^2^	mm/rad^2^	mm/rad	mm/rad^2^	mm/rad	/
$2.4984\times 10^{-7}$	6.9608×	$2.9786\times 10^{-9}$	$-6.3406\times 10^{-5}$	$1.5465\times 10^{-6}$	$-2.3959\times 10^{-7}$	$-6.6406\times 10^{-5}$	$5.1507\times 10^{-6}$	$2.9618\times 10^{-5}$	$8.0886\times 10^{-3}$

**Table 5:** Settings of the LLS.

Setting	Value
Line distance.	0.25 mm
Point distance	0.01 mm
Laser power	100%
Exposure	15%
Signal threshold	10%
3D filtering	Off
Edge filter	5
Reflectivity filter	On

**Table 6:** The second validation test with calibrated diameter, measured results and the estimated confidence interval.

Calibrated Diameter/mm	*LCL*/mm	Measured Diameter/mm	*UCL*/mm	Validated?
60.0010	60.0004	60.0016	60.0029	✓
70.0020	70.0007	70.0019	70.0031	✓
89.9969	89.9955	89.9968	89.9981	✓
125.0050	125.0034	125.0048	125.0062	✓
149.9994	149.9966	149.9983	150.0001	✓

## Data Availability

The data that support the findings of this study are available upon reasonable request from the authors.
